# The economic impacts of foot and mouth disease – What are they, how big are they and where do they occur?

**DOI:** 10.1016/j.prevetmed.2013.07.013

**Published:** 2013-11-01

**Authors:** T.J.D. Knight-Jones, J. Rushton

**Affiliations:** a The Pirbright Institute, Ash Road, Pirbright, Surrey GU24 0NF, United Kingdom; b The Royal Veterinary College (VEEPH), Hawkshead Road, North Mymms, Hertfordshire AL9 7TA, United Kingdom

**Keywords:** Economics, FMD, Review, Impact

## Abstract

Although a disease of low mortality, the global impact of foot and mouth disease (FMD) is colossal due to the huge numbers of animals affected. This impact can be separated into two components: (1) direct losses due to reduced production and changes in herd structure; and (2) indirect losses caused by costs of FMD control, poor access to markets and limited use of improved production technologies. This paper estimates that annual impact of FMD in terms of visible production losses and vaccination in endemic regions alone amount to between US$6.5 and 21 billion. In addition, outbreaks in FMD free countries and zones cause losses of >US$1.5 billion a year.

FMD impacts are not the same throughout the world:

1. FMD production losses have a big impact on the world's poorest where more people are directly dependent on livestock. FMD reduces herd fertility leading to less efficient herd structures and discourages the use of FMD susceptible, high productivity breeds. Overall the direct losses limit livestock productivity affecting food security.
2. In countries with ongoing control programmes, FMD control and management creates large costs. These control programmes are often difficult to discontinue due to risks of new FMD incursion.
3. The presence, or even threat, of FMD prevents access to lucrative international markets.
4. In FMD free countries outbreaks occur periodically and the costs involved in regaining free status have been enormous.

FMD is highly contagious and the actions of one farmer affect the risk of FMD occurring on other holdings; thus sizeable externalities are generated. Control therefore requires coordination within and between countries. These externalities imply that FMD control produces a significant amount of public goods, justifying the need for national and international public investment.

Equipping poor countries with the tools needed to control FMD will involve the long term development of state veterinary services that in turn will deliver wider benefits to a nation including the control of other livestock diseases.

## 1 Introduction

**Fig. 1:**
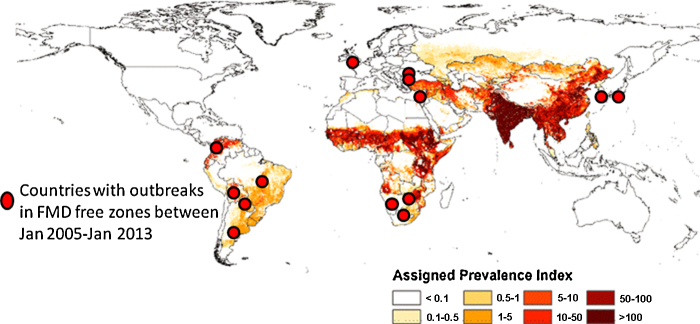
Global burden of FMD in cattle; burden of FMD in sheep and goats had a similar distribution. Measured as a prevalence score based on estimates of incidence, population distribution and other risk factors, adapted from Sumption et al. (2008).

Foot and mouth disease (FMD) has been eradicated by many wealthy nations but remains endemic in most of the world (see Fig. 1). When FMD outbreaks occur in disease free countries and zones that produce livestock for export the economic impact is clear to see; however, the impact of the disease in endemic countries is more controversial, particularly when compared to diseases that cause greater mortality.

In recent times there has been increased consideration of FMD control in endemic countries. Knowledge of disease impact is essential when deciding on the level of expenditure that can be justified by a disease control programme. Impact, together with the marginal returns for investing in disease control should be compared for different diseases, considering the cost of control measures and their likely effect.

There is always a danger that conclusions on disease impact will be based on observations of affected individuals or farms, particularly if losses are dramatic. When considering the burden of a disease one must step back and consider its impact at the population level. To consider this as a function of losses in diseased individuals and the number affected is an over-simplification; for livestock diseases and FMD in particular the full impact of a disease is far more complex.

Although FMD is a disease of low mortality the frequency of outbreaks and the large numbers of animals and species affected in each outbreak results in a high and on-going impact for FMD in endemic countries (Onono et al., 2013). FMD endemic countries collectively contain three-quarters of the world's population (Thornton et al., 2002; Robinson et al., 2011).

Livestock movements and trade play a key role in the spread of FMD. Hence, despite the significant economic losses involved (James and Rushton, 2002), movement and trade restrictions at domestic and international level are fundamental to control (Sutmoller et al., 2003).

The objective of this paper is to describe the economic impact of FMD including how it varies in different settings and how knowledge of this should be used to guide control policy. This included a synthesis of current literature on the subject. To help appreciate the scale of global FMD impact estimates were made of the direct costs of disease and vaccination in endemic countries as well as outbreak costs in free countries.

## 2 Literature review

### 2.1 Methods

The literature search covered published journal articles, reports and grey literature and was performed using the following methods:

(a) Online search; ProMED, google scholar and google web were searched for papers with “FMD” or “foot and mouth disease” and “economic*” or “impact” or “cost-benefit”.
(b) Eight experts in the field of FMD economics were asked to provide suitable publications.
(c) References of interest in identified papers were reviewed.
(d) Other relevant publications that the authors were aware of were included.

Articles written in English or Spanish were included. Articles were retained if they reported either original research or reviewed aspects of FMD economic impact. Articles reporting the predicted impact or cost-benefit of future FMD control or of FMD control already conducted during an outbreak are presented in a separate section.

### 2.2 Categorised impacts of FMD

**Fig. 2:**
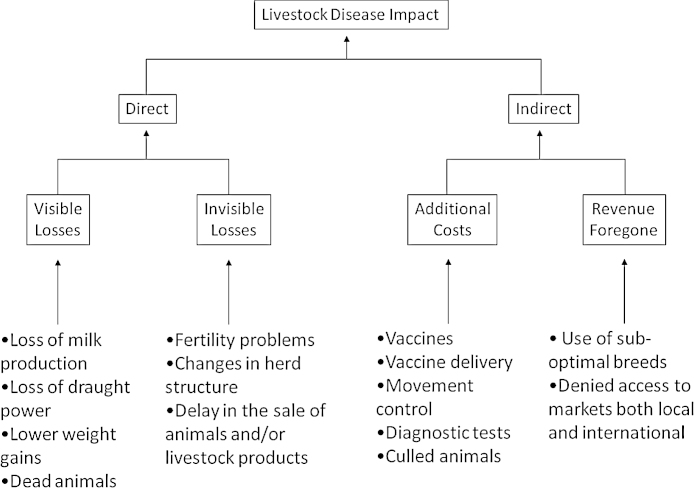
The impacts of foot-mouth-disease (Rushton, 2009).

FMD affects all the major non-avian livestock species causing high morbidity and low mortality, although high mortality of young stock can occur (James and Rushton, 2002; Perry and Randolph, 2003; Perry and Rich, 2007; Perry and Sones, 2007; Perry and Grace, 2009). Fig. 2 shows the different impacts of FMD (Rushton et al., 1999; Rushton, 2009). The framework used differs to the one proposed for livestock diseases by McInerney et al. (1992), and McInerney (1988,1996).

## 3 Direct impacts

### 3.1 Visible losses

Production losses due directly to FMD include reduced milk production (Bayissa et al., 2011), affecting both the humans and calves that depend on it. This can account for 33% of losses in endemic settings (Ellis and Putt, 1981). Not only crucial to commercial dairy operations, milk is an important source of nutrition for many pastoralists, particularly for children (Barasa et al., 2008). Although FMD typically has a short-term affect on an animal's health, chronic FMD typically reduces milk yields by 80% (Bulman and Terrazas, 1976; Barasa et al., 2008; Bayissa et al., 2011). Livestock growth rates are also suppressed and mortality amongst young stock is typically 2–3% (Rufael et al., 2008) although occasionally much higher (OIE/Iowa State University, 2007; Barasa et al., 2008). Loss of traction power where draught animals are used is particularly damaging if it occurs during harvest (Ellis and James, 1976; Perry et al., 1999; Perry and Randolph, 2003). FMD can result in abortion, the cost of which is high as the farmer will have to pay to keep the cow without it producing anything for another year or more, or cull the animal.

Visible production losses are most prominent in pigs in intensive production systems and dairy cattle. These two systems are key sources of animal protein in poor countries and their importance continues to grow (Delgado et al., 1999).

### 3.2 Invisible losses

A compound effect of fertility problems due to abortion and reduced conception rates is a need to have a greater proportion of breeding animals in a population for a given output. This invisible loss means that for every kilo of meat or milk produced there is an additional fixed cost to maintain more breeding stock (Rushton, 2009).

## 4 Indirect impacts

### 4.1 Additional costs

#### 4.1.1 Control costs

The cost of control carried out by the state veterinary services (e.g. vaccination, outbreak control, culling and compensation) is borne by the tax payer. In addition significant amounts are spent by the private sector. These costs are enormous with an estimated 2.35 billion doses of FMD vaccine administered in the world every year (Table 1) (Hamond, 2011) at a cost of $0.4–3 or occasionally $9 per dose including delivery and application (Sutmoller et al., 2003; Barasa et al., 2008; Forman et al., 2009). Due to the short duration of immunity induced by FMD vaccines, ongoing control programmes vaccinate cattle one to five times a year and sheep and goats once a year; limiting resources available to combat other diseases.

Wildlife are sometimes kept out of FMD free zones with fencing which is both costly and affects wildlife ecology (Gadd, 2011).

Even if a country is FMD free there are ongoing costs due to efforts to prevent disease introduction, including import controls and sometimes vaccination. In addition, maintaining FMD early detection and control capability, including vaccine banks, is costly. Other costs include FMD related research and permanent restrictions on the livestock sector (such as post-movement standstills and bans on feeding swill).

The cost of surveillance are significant, including proving disease freedom after an outbreak; >3 million serum samples were tested after the UK 2001 outbreak (Paton et al., 2006) in addition to approximately 3.5 million sera tested during the outbreak.

Control measures can affect other industries, a worst case example being the UK 2001 outbreak which caused US$4–5 billion in lost tourism revenue (Thompson et al., 2002). Culling based control measures can have wider impacts including public outrage, depression and suicides amongst farmers (Mort et al., 2005), pollution from carcasses and animal welfare issues. Movement restrictions disrupt the normal flows of animals between different units and enterprises at different stages of their life and can result in welfare problems if access to housing and grazing is prevented; in the UK 2001 outbreak welfare reasons accounted for one third of animals culled (Mansley et al., 2011).

### 4.2 Revenue foregone

#### 4.2.1 Market access

Countries infected with FMD cannot trade live animals with FMD free countries. Typically the countries with the best meat prices are FMD free (i.e. EU, USA and Japan) (James and Rushton, 2002) where prices are typically 50% higher (Jarvis et al., 2005).

The trade of livestock products is also restricted. If regular outbreaks occur only processed, tinned products can be exported to free countries; if FMD is effectively controlled with vaccination by a competent veterinary service able to detect outbreaks then deboned meat can be exported (James and Rushton, 2002). Also, trade of fruit and vegetables can be affected by FMD status (James and Rushton, 2002). Even if a country is FMD free, if it trades with FMD infected countries it will experience trade restrictions (James and Rushton, 2002).

Lack of access to lucrative markets has further consequences; it restricts the development of commercial farming. Restrictions limit the supply of livestock and livestock products to free countries; although this is good for domestic producers it leads to increased market prices for consumers. If FMD free status is lost livestock are dumped on the domestic market, reducing prices for consumers at the cost of producers. Even within an endemic country livestock trade is limited; those affected by FMD receive lower prices for their stock and those wishing to purchase animals from FMD free herds face a restricted supply. Furthermore, investment in the livestock sector is limited if there is a perceived risk that FMD may occur. High productivity breeds are typically more susceptible to FMD. The threat of FMD therefore restricts (a) the use of these breeds and (b) prevents the development of more intensive production.

#### 4.2.2 Externalities

FMD is highly contagious, affects many species and is not easily contained within one farm or one population. The presence of FMD creates problems to all livestock owners who are connected to populations where FMD is present. This connection may be geographical or via market chains. Therefore, FMD creates what economists call externalities. If an outbreak occurs because one farmer did not protect his animals others may suffer. Conversely when a livestock owner protects their animals from FMD infection they will generate a positive externality as they are less likely to become infected and transmit the pathogen to other farms.

The positive and negative impacts of FMD on different players in a dynamic market are complex; when FMD outbreaks create increased demand for vaccines, pharmaceutical companies benefit. When a free country experiences an outbreak poultry prices may increase due to public reluctance to consume products from FMD susceptible species, particularly if through ignorance there is a reluctance to eat products from FMD vaccinated animals.

Where externalities exist there is a need for public investment as one farmer's actions create costs and benefits for others. These externalities are not equally shared amongst different livestock sectors (Perry and Randolph, 2003) with production losses being particularly severe for commercial dairy farms. Even when individuals reap positive returns from successful FMD control there is less of an incentive to undertake such a programme if there is a high risk of reinfection from those that do not attempt FMD control.

Effective control of infectious diseases with vaccination often requires high levels of vaccine coverage to develop herd immunity; with a sufficient proportion of immune animals outbreaks will tend to die out due to a lack of susceptible hosts. If left in the hands of individual farmers a lack of action by those less visibly affected by FMD will result in pockets where control is poor, undermining the entire control programme. Impacts on the livestock producer have ripple effects along the entire market chain, impacting on other players, such as markets, abattoirs and dairies to mention a few (Le Gall and Leboucq, 2004). FMD control can be both an externality, with benefits not captured by the market, and a regional or global public good, as the reduction in risk of FMD is also experienced by countries other than ones controlling the disease; external funding and cooperation is therefore required (Forman et al., 2009).

#### 4.2.3 FMD impact in different countries

The impact of disease is not equal across all countries and livestock populations due to differences in not only FMD status, incidence and risk of incursion but also (a) the genetics of the national herd; (b) prevailing livestock management practices; (c) prevailing prices of livestock production inputs and outputs (Rushton, 2009) and (d) their ability to supply livestock for export markets. This is easier to appreciate when one considers specific countries which differ in these characteristics.

(i) *The impact of FMD in a disease free country with export potential but where FMD is present in the wider region.*
  In this setting the main impacts are through the cost of on-going control, particularly vaccination, and loss of export markets and further control measures when outbreaks occur.
  *Example 1 – Uruguay*
  The case of Uruguay highlights the benefits of FMD control, particularly if it allows export markets to open up. Upon gaining free status without vaccination in 1996 the value of exports increased by over 50%, providing an added $110 million of revenue per year through exports to America, consisting of 20,000 tonnes of beef sold at double domestic prices; in addition there was increased trade with the Pacific rim countries (Otte et al., 2004). Savings of $8–9 million per year were initially made via avoided routine vaccination costs, however, after an outbreak in 2001 ongoing vaccination was re-introduced due to the threat of infection from neighbouring countries (Sutmoller et al., 2003). Controlling the 2001 outbreak cost $13.6 million, 55% of which was due to vaccination costs. This included two rounds of nationwide mass vaccination for 10.6 million cattle; 24 million vaccine doses were used, mostly administered by farmers. The remaining 45% of costs were for farmer compensation, cleaning and disinfection, and operating costs. The estimated total cost of the outbreak (including loss of trade) was US$700 million (Personal Communication, F. Munzio).
(ii) *The impact of FMD in a disease free country with significant livestock exports and relatively low risk of incursion.*
  In this setting the major impact is through maintaining preparedness due to the dire economic consequences of an FMD incursion.
  *Example 2 – Australia*
  In 2000–2001, the gross value of Australian livestock industry was US$8 billion with exports worth US$7.5 billion. FMD control costs include maintenance of preparedness and efforts to keep the virus out or minimise the impact should an outbreak occur. Australia also funds FMD control in countries most likely to act as a source of any future virus incursion into Australia. Due to reduced prices for export products in non-FMD free markets, loss of FMD free status could cause Australian export revenue to fall by 70% in the first year after the outbreak (Garner et al., 2002). Increased supply to the domestic market would reduce meat prices, which although beneficial for the consumer would lead to additional industry losses of >US$1.5 billion for a 12 month outbreak with total losses for such an outbreak falling between US$4 and 6.5 billion (converted using 2001 exchange rate where AUS$1 = 0.5US$). As long as it remains FMD free, Australia benefits from FMD being present in other countries as it reduces competition for lucrative export markets, resulting in high prices for their exports (Productivity Commission, 2002).
(iii) *The impact of FMD in a disease free country which imports livestock products.*
  In this setting the major impact is due to the high price paid for importing meat from FMD free countries only. Other ongoing control costs may also exist.
  *Example 3 – Indonesia*
  As the archipelago of Indonesia is FMD free the disease has no impact on production and ongoing vaccination is not required. However, the country imports significant amounts of meat. To reduce the chance of virus incursion Indonesia only imports meat from FMD free countries like Australia. The price of meat from these sources is much higher. This is essentially an additional FMD control cost paid to reduce the risk of FMD virus being imported. Within Indonesia growing income levels have increased the demand for meat causing further increases in the price of meat. High beef prices have resulted in increasing quantities of meat being smuggled into the country from India where FMD is present but where meat is cheaper. To reduce the price of legal meat imports the government is considering relaxing regulations to allow imports from not only FMD free countries but also FMD free zones in countries with endemic regions (such as Brazil).
(iv) *The impact of FMD in an endemic country with limited export potential looking to increase national productivity and reduce risk to neighbouring countries.*
  In this situation the main impacts are disease-induced production losses, ongoing vaccination costs, premium prices paid for FMD free imports and the risk the country poses to neighbouring free countries.
  *Example 4 – Turkey*
  FMD is endemic in most of Turkey, however, a free zone with vaccination exists in the region in continental Europe (Thrace). Great efforts have been made to control FMD in Turkey via vaccination. Turkey acts as a FMD virus reservoir which could spill over into disease free Europe. To combat this the European Commission spent €65 million on vaccination in endemic Turkey between 2008 and 2011 (Sumption, 2009) with additional ongoing funding for vaccination in the free zone.
  FMD is widespread with approximately 10% of the 11 million cattle population infected with FMD before the age of 2 years (Askaroglu, 2009) and the direct impact of FMD for each case costing on average $150–300 depending on production type [i.e. dairy, beef, dual purpose] (Şentürk et al., 2008). Limited domestic production led to Turkey's meat prices being amongst the highest in the world; in 2010 beef was over €8/KG and yearling Friesian bulls sold for €1500. Importation of live cattle was highly restricted in part to reduce the chance of importing diseases such as Bovine Spongiform Encephalopathy; in 2010 very few countries could legally export live cattle to Turkey (McCarthy, 2010). High prices encouraged illegal imports from neighbouring FMD endemic countries which in turn undermined FMD control in Turkey.
  More countries can now export to Turkey and import taxes have been reduced. Even countries that export livestock to countries other than Turkey, benefit from trade rivals diverting their exports to Turkey as this reduces supply elsewhere resulting in higher prices.
(v) *The impact of FMD in an endemic country with the potential to export.*
  In a country like this the control costs required to attain and maintain free status are sizeable and the risk of subsequent outbreaks in free zones may be high. If FMD free trade can be established the benefits are significant, however, other barriers to market access may exist.
  *Example 5 – Zimbabwe*
  Zimbabwe had a zonal FMD control programme based on wildlife control and vaccination much like Botswana, Namibia and South Africa have today. Until 2007 Zimbabwe could export meat to the EU with reduced import tariffs. This trade brought in $50 million per year up to 2001 (Scoones and Wolmer, 2007). In 2003 Perry et al. (2003) estimated that if FMD control was reduced, every dollar saved in reduced control costs would result in $5 lost to the national economy. The breakdown of FMD control in Zimbabwe after the land reforms of 2000 has also had a big impact on Botswana where spill-over FMD outbreaks have jeopardised the valuable beef export industry.
  FMD control in Zimbabwe was largely funded by the tax payer and external donors. Although FMD control measures impacted on all livestock owners, the benefits were largely received by a relatively wealthy minority involved in the commercial beef sector. If FMD free status was regained it would take time to re-establish a beef industry capable of supplying a sufficient quantity of export quality beef.
  *Example 6 – Ethiopia*
  Ethiopia has the largest cattle population in Africa; in 2006 there were >43 million cattle with slightly fewer sheep and goats (Rich et al., 2009). Large numbers of ruminants are exported; in the Ethiopian financial year (July 2010–July 2011), meat and livestock export revenue was $211.1 million, mostly from live animal trade with the Middle East (>472,041 heads of live animals, 70% of which were cattle) (SPS-LMM, 2011). However, production costs are high compared to other meat exporting nations, such as Australia or Brazil, limiting the potential for export market access regardless of FMD status. Difficulties in meeting export Sanitary and Phyto-Sanitary standards results in greater numbers of livestock being purchased by traders for export through unofficial channels where prices are lower.
  Due to the presence of FMD and other OIE listed trade limiting diseases the export of live cattle and their products to FMD free countries is an unlikely prospect (Rich et al., 2009). This raises the case for investment in veterinary service infrastructure to improve the control of all trade limiting diseases for international market access.
  Having an economy that is highly dependent on smallholder and animal-based agriculture, including the widespread use of beasts of burden, the direct impacts of FMD are substantial in Ethiopia. In agro-pastoral areas, FMD infected oxen are unable to work for the entire season when affected at cropping time. Pastoralists are particularly vulnerable to FMD as their living depends entirely on their livestock (Bayissa et al., 2011). By reducing the supply of milk FMD impacts on food security, particularly when outbreaks occur during times of the year when other food sources are limited and dependency upon milk is greatest (Barasa et al., 2008).

### 4.3 FMD control

#### 4.3.1 Control: the rationale

If money is spent on disease control, the intention is to reduce losses elsewhere by a greater amount. These losses may be due to reduced production or restricted market access. To control FMD governments must create an environment where population level control costs reflect the benefits experienced by the livestock sector and the wider economy. This requires a combination of:

• Investments in veterinary services, education, research and general infrastructure to develop the animal health system – what economists would call fixed costs.
• Specific programmes that cover the costs of FMD control and management – what economists would call variable costs.

In many countries there is already a fixed cost investment in animal health systems, and adding an FMD control programme is relatively easy. However, countries that have low level investments in animal health will struggle to implement an effective FMD control programme. In this situation there needs to be an increase in both the fixed and variable costs. The fixed cost element will generate capacity and skills that will benefit the control of other diseases and therefore not all costs for this element should be assigned to FMD.

#### 4.3.2 Control: the reality

FMD transmission is controlled by both reducing an animal's chance of virus exposure and reducing susceptibility via vaccination or culling high risk animals in case virus exposure cannot be prevented. Socio-economic factors influence both aspects.

Control measures such as movement restrictions and culling create hardship. To encourage individual self-sacrifice for public disease control the carrot and stick of compensation and enforcement are required. Unfortunately production types with the least interest in FMD control are often more prevalent in countries least able to compensate and enforce. This is self-perpetuating; as if FMD is not controlled farmers will keep animals less susceptible to FMD to reduce its visible impact increasing the need for compensation and enforcement, yet high incidence and limited public budget makes this unfeasible. Indirect losses due to restricted market access are less tangible to less commercially driven farmers and if other trade barriers (including other diseases) are present the benefits of FMD control become more distant.

A sensible approach to increasing global FMD control would be to identify areas where control is most likely to succeed such as regions with motivated, commercial farms and low farm density. Small holder systems are problematic as they often have extensive between farm contacts through high farm densities, frequent trading and a dependency on communal grazing. In addition they may have fewer visible incentives to control FMD. Small holdings are logistically difficult to vaccinate with high coverage and they are more prevalent in countries with less effective veterinary services unable to enforce restrictions and to compensate.

If widespread control is not possible, farmers keen to control FMD should be assisted by making quality vaccines available (if efficacy is questioned uptake will be low) and limiting the damage of those not participating in FMD control. Some countries fully subsidise vaccination in small holdings, funded by governments and industry bodies, whilst larger farms pay for vaccination. Although not yet an option, commodity based trade would allow enterprises to access lucrative markets without the enormous hurdle of achieving national or zonal freedom (Rich and Perry, 2011).

## 5 Global impact

### 5.1 Economic analyses of FMD and its control

The preferred method of estimating global FMD impact would be to aggregate national and regional studies. This is the approach taken by the World Health Organization when estimating global disease burdens (Senior, 2009).

The literature was searched for studies of FMD economic impact. There has been no study carried out for a global strategy for FMD control and eradication, but just over 30 country or regional studies have been published in the peer reviewed and grey literature (see Table 2). A large number of these are ex post evaluations after large outbreaks in previously free countries. Countries that are free have also carried out a number of outbreak simulations studies. Finally there is a limited set of studies on FMD control in endemic countries. The challenges and uncertainties associated with FMD control in endemic regions are considerable; predicting returns on investment in FMD control in these settings is not easy. Aggregating these papers and filling in the gaps to incorporate them into a global estimate is beyond the scope of this paper but this literature synthesis reveals the following:

• Control programmes in countries previously free generate positive returns to the economy.
• Countries free from FMD that suffer an outbreak lose between 0.2% and 0.6% of GDP.
• In countries with international trade in livestock and livestock products the control of FMD has good economic returns.
• In countries with limited or no international trade in livestock and livestock products a positive return on FMD control requires targeted programmes.
• There is a lack of studies that examine the full economic cost of FMD in endemic countries particularly considering indirect losses at the national level.

There has also been very limited work carried out on the economic analysis of farm-level control of FMD, an important consideration in the success of disease control. Both Ellis and James (1976) and Bulman and Terrazas (1976) indicate high impact of FMD and positive returns to its control for dairy systems in India and Bolivia, respectively. Rushton et al. (2002) indicate that FMD would have a high impact in dairy and pig systems, but limited or no impact on sheep and beef systems. In Bolivia a study indicated that there was no positive return to farm-level control of FMD with preventive vaccination (Rushton, 2008).

Five recent studies on FMD impact on small holder systems were identified. Studies in Cambodia reported a reduction in household income of 4.4–11.7% annually following an outbreak of FMD with a loss of 54–92% of animal value (Shankar et al., 2012; Young et al., 2012). This compares to a reported 22–30% loss in of animal value in Laos following FMD (Rast et al., 2010). In Sudan losses of US$25 per cow per year were found in a region where 90% of the population have an income of less than 1 dollar a day (Barasa et al., 2008). Finally a study in Pakistan found that compared to pre-FMD milk yield 60 days after disease was still reduced by a third (Ferrari et al., 2013); in this study vaccination had an estimated cost/benefit ratio of 5.7, this compares to 11.5 in South Sudanese pastoralists (Barasa et al., 2008).

### 5.2 The magnitude of global impact – endemic regions

To demonstrate the scale and distribution of the global FMD burden we have made an approximate estimate of the impact of direct losses and vaccination due to FMD in endemic countries. These impacts fall on affected farmers and those that pay for vaccination, usually the state. A more complex analysis considering externalities and knock on effects on market prices and how this affects different producers in different countries and sectors is beyond the scope of this paper. Calculations were made using the statistical software R (R Development Core Team, 2010) using Monte-Carlo simulation with 20,000 simulations.

#### 5.2.1 Numbers of animals affected

**Fig. 3:**
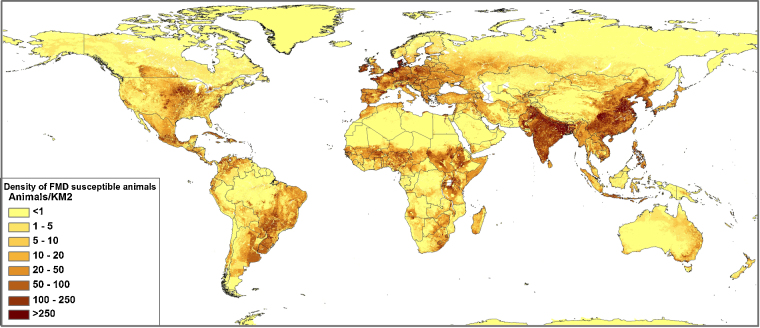
Density map of FMD susceptible livestock species, i.e. combined cattle, pigs, sheep and goats (Wint and Robinson, 2007; Di Nardo et al., 2011).

Based on estimates of FMD incidence and population size (FAO stat gridded livestock population (Wint and Robinson, 2007) – see Fig. 3) an estimate was made of the number of animals affected by FMD in a year with the current control measures in place. This was done for all countries using the nine incidence categories in Sumption et al. (2008). Results were then aggregated by region (see Table 3).

The numbers of animals were converted to livestock units to estimate the economic value of livestock affected on a yearly basis (i.e. one LSU = 1 head of cattle, 3.3 pigs or 10 sheep or goats).

To account for uncertainty of the estimate, incidence was multiplied by an uncertainty factor. This factor was thought to range from a minimum and most likely value of 1 (i.e. the incidence estimate was correct) to 8 (i.e. only one in eight cases were accounted for), described by a Betapert distribution (min = 1, max = 8, mode = 1). That is, in many cases the estimates in Sumption et al. (2008) are the best estimates available. However, it could be that in some instances the original estimate used was an eighth of the true incidence; the latter figure was derived from work done in Iran, comparing incidence of reported cases to incidence of infected premises detected by serology (Emami et al., 2012). Iran was used in Sumption et al. (2008) to derive incidence in endemic countries for which little information was available assuming under-reporting was uncommon in Iran.

It was estimated that 32 million livestock units (LSU) are affected by FMD in a year, although the figure could be between 28 and 79 million (5th and 95th percentile of the uncertainty distribution, median = 41 million). The worst affected regions in terms of absolute numbers are China, Africa and India (see Table 3).

Three quarters of the livestock units affected by FMD are predicted to be cattle and 13% pigs. The impact on cattle is greatest in Africa, India, rest of Asia and China, whereas the impact of the disease in pigs is estimated to be greatest in China. In terms of the proportion of livestock affected we estimate that around 2% of the world's cattle population has FMD in a year (90% uncertainty range: 2–5%), but there are regional differences with China and India the worst affected areas (see Table 3).

Some regions have FMD strains that cause greater impact in certain species, so for example a pig adapted strain will have a greater impact when present in an area of high pig density; this level of complexity was not modelled.

#### 5.2.2 Number of vaccine doses

Estimates made by leading FMD vaccine manufacturers of the number of vaccine doses produced per region in 2010 were used (see Table 1).

#### 5.2.3 Direct impact losses and vaccine costs

The overall economic impact was calculated based on the costs of a vaccine and its application being between US$0.4 and US$3, with most likely cost US$1 [triangle distribution (min = 0.4, max = 3, mode = 1)] (Sutmoller et al., 2003; Barasa et al., 2008; Forman et al., 2009). Direct, visible FMD production losses were assummed to range from US$100 to US$370 per case, with the most likely value US$100 [triangle distribution (min = 100, max = 370, mode = 100)] (Barasa et al., 2008; Şentürk et al., 2008; Shankar et al., 2012; Ferrari et al., 2013). The latter estimate takes into account the death of an animal, loss in weight gain, milk production and draught power. This was multiplied by the number of cases per year to estimate total direct losses.

The total annual impact of FMD due to direct losses and vaccination was estimated to range between US$6.5 billion and US$21 billion (90% range) with an average value of US$ 11 billion (see Table 4). The majority of FMD impact occurs in China, India and Africa. In Africa it has been estimated that more is spent controlling FMD than any other veterinary disease (Le Gall and Leboucq, 2004). Impact in South America is largely due to the costs of vaccination.

#### 5.2.4 Limitations

The impact estimate does not account for restricted development of the livestock sector when FMD is present or other FMD control and surveillance costs. In addition it does not include the losses due to trade restrictions which although large at both the local and international level could not be estimated with any accuracy and are highly variable. Even when focussing on the most visible and tangible impacts of FMD, estimates are uncertain with wide confidence intervals. Therefore US$11 billion quantifies some but not all aspects of FMD impact in endemic countries.

One study estimated that FMD losses due to deaths, milk loss, draught losses and treatment costs were US$2.7–3.6 billion in India alone (Ganesh Kumar, 2012).

### 5.3 Outbreaks in FMD free countries

The above gives an indication of the ongoing burden of FMD in endemic countries, but outbreaks in free countries can be devastating. In the country examples in Table 5, these outbreaks cost from half to almost 10 billion US dollars. In 2011 and 2012 there were 10 reported outbreaks in FMD free zones or countries (OIE, 2013).

The 2001 UK FMD outbreak highlights how severe and widespread the consequences of an outbreak in an FMD free country can be. Widespread culling was used to contain the disease and ultimately over 6 million animals were slaughtered, approximately 7% of all UK cattle and 15% of all sheep (Rushton et al., 2002).

In recent times Asia has suffered major FMD epidemics in countries that were previously free. In Taiwan an outbreak of FMD (1997), mainly in the pig population, decimated the sector and was estimated to have reduced the total GDP of the country by 0.28% (Hsu et al., 2005). Japan has had FMD outbreaks in 2000 and 2010 (Muroga et al., 2012), and the Republic of Korea experienced an outbreak in 2010 and 2011 with the destruction of 3.4 million livestock and costs of US$ 2.78 billion (ProMED/Yonhap news agency, 2011; FAO, 2012).

In total over US$20 billion has been lost during the last 15 years due to major FMD epidemics in countries that were previously free (US$25 billion if corrected to the value of a US$ in 2011); this equates to about US$1.5 billion per year. In addition there were many smaller outbreaks (see Fig. 1) in FMD free zones or countries; although the economic impact of these outbreaks is small compared to the major outbreaks in Table 5, the amounts are still significant [e.g. the UK 2007 outbreak involved eight farms and cost the government GB£47 million and industry GB£100 million, i.e. total US$300 million (Anderson, 2008)]. If one considers these figures in conjunction with the US$6.5–21 billion vaccine costs and direct losses in endemic countries one gets some idea of the magnitude of global FMD impact.

## 6 Conclusion

Wealthy countries that have eradicated FMD face ongoing costs from periodic outbreaks and the costs of maintaining preparedness. Many countries reduce the impact of the disease with extensive ongoing vaccination programmes. The global scale and costs associated with these programmes is vast with billions of doses administered annually. Restricted access to international markets due to FMD greatly reduces revenue for nations with the capacity to export livestock and their products.

The impact of FMD in endemic countries has received less attention than the impact of outbreaks in free countries despite the huge numbers of animals affected and the importance of livestock in these countries (see Table 3). Direct losses due to death and disease are easy to appreciate, however, in endemic countries the burden of FMD often manifests as widespread and ongoing losses that limit development of the livestock sector. FMD impacts on different enterprises and countries in different ways; the consequences of this variable impact and risk should be considered when planning disease control.

## Figures and Tables

**Table 1:** Estimated FMD vaccinations by country per year [based on the number of vaccine doses produced, as estimated by leading FMD vaccine manufacturers using expert opinion and industry data (Hamond, 2011)] and the population targeted (based on author's consultations and Wint and Robinson (2007)).

Region	Vaccinations		Population targeted		
	Doses (millions)	%	Species	Population (millions)	% vaccinated^a^
China	1600	68.1	Cattle, shoats, pigs and buffalo	833	192.2
India	150	6.4	Cattle and buffalo	280	53.6
Rest of Asia	50	2.1	Cattle, pigs and buffalo	283	17.7
Africa	15	0.6	Cattle	272	5.5
Europe and Turkey	15	0.6	Cattle	140	10.7
Middle East	20	0.9	Cattle and shoats	167	12.0
South America	500	21.3	Cattle	342	146.1
Total	2350	100.0		2036	115.4

a Calculated as the number of vaccine doses × 100/population size; values >100% imply that on average animals were vaccinated more than once a year.

**Table 2:** Cost benefit analysis studies of FMD control and eradication programmes.

Country	Export potential	Returns to control	Analysis	Author
Australia	Large	A 6 month outbreak would reduce GDP by 0.6%	Simulation	Garner et al. (2002)
Australia	Large	Losses to the national economy of $2–3 billion or $8–13 billion can be expected depending on outbreak length	Simulation	Productivity Commission (2002)
Bhutan	Nil	Positive when control focused on endemic areas, negative if unfocussed	Data analysis	Tshering (1995)
Bolivia	Small	Negative, analysis was based on a prolonged programme and reliable data	Data analysis	FAO (1995)
Bolivia	Small	Positive, but with a short intensive vaccination campaign in the endemic areas	Data analysis	PANAFTOSA (1997)
Bolivia	Small	Positive, but control of FMD is not economic for extensive systems, hence greater public funding is required	Data analysis	Rushton (2008)
Botswana	Large	Positive with exports, negative without exports	Data analysis	Oarabile (1994)
Canada	Large	Even a small outbreak could cost $2 billion over 5 years	Simulation	Krystynak and Charlebois (1987)
France	Large	Rapidly regaining export market access is key, this is best achieved by stamping out	Simulation	Mahul and Durand (2000)
UK	At that time small	Positive for both a stamping out policy and for vaccination	Data analysis	Power and Harris (1973)
India	Small	Positive due to the large returns in the milk sector	Data analysis	Ellis and James (1976)
Netherlands	Large	Culling is preferable in areas of low livestock density, vaccination is preferable in areas of high density. Market acceptance of products from FMD vaccinated animals reduces the impact of an outbreak	Simulation	Backer et al. (2009)
Netherlands	Large	The 2001 FMD outbreak cost the nation €1billion	Data analysis	Huirne et al. (2002)
New Zealand	Large	An outbreak could cost $NZ10 billion, with eradication by slaughter being preferable to vaccinate to live	Simulation	Belton (2004)
Philippines	Unknown	Positive, particularly benefiting the commercial pig sector. Benefit-cost ratio of 1.6–12 depending on level of exports	Data analysis	Randolph et al. (2002)
Sudan	Nil	Positive with increased food security. Benefit-cost ratio of 11.5 with successful vaccination	Data analysis	Barasa et al. (2008)
Southern Cone	Large	Positive for both culling and vaccination strategies, does not deal with social impacts and feasibility of implementation	Data analysis + simulation	Rich and Winter-Nelson (2007)
Taiwan	Large (pig products to Japan)	Returns according to the information on eradication are large with costs of eradicating the 1997 outbreak estimated to be US$378.9 million, but with potential export losses of approx. US$1.2 billion	Data analysis	Yang et al. (1999)
Taiwan	Large	Losses due to the 1997 FMD outbreak were experienced in many sectors, causing a 0.28% loss to GDP	Data analysis	Hsu et al. (2005)
Thailand	Possible	Positive with a benefit cost ratio of 3.73 and 15 with and without export of livestock products respectively	Data analysis	Perry et al. (1999)
Turkey	Unknown	Culling certain highly susceptible cattle could be viable	Data analysis	Şentürk et al. (2008)
UK	Large	The lowest cost strategy comparing vaccination to culling depended on other factors, such as outbreak size	Simulation	Risk_Solutions (2005)
UK	Large	Vaccination may not be the most effective way of controlling an outbreak, however, speed of regaining export market access is not the only consideration	Data analysis	Rushton et al. (2002)
UK	Large	GDP fell by less than 0.2% due to the 2001 FMD outbreak	Data analysis	Thompson et al. (2002)
USA	Large	Vaccination based eradication provides the best return when the vaccine is effective	Simulation	Bates et al. (2003)
USA	Large	If time to outbreak detection extends beyond 21 days, every additional hour delay results in extra losses in the order of $565 million	Simulation	Carpenter et al. (2011)
USA	Large	A large FMD outbreak could lead to a $14 billion loss in farm income, with loss of exports and fall in demand due to consumer fears	Data analysis	Paarlberg et al. (2002)
Uruguay	Strong	Control brings strong positive returns based on the access to export markets (20,000 tonnes beef export to USA)	Data analysis	Leslie et al. (1997)
Southern Africa	Strong at that time	Positive benefit, particularly for commercial farms, less so for the poor. Every dollar saved on control leads to $5 lost to the economy	Data analysis	Perry et al. (2003) and Randolph et al. (2005)

**Table 3:** Estimated number and cumulative incidence (%) of livestock affected by FMD per year by region. Estimated using FAOSTAT population estimates (Wint and Robinson, 2007) and incidence estimates from Sumption et al. (2008). Outbreaks in free countries excluded. Original estimates are shown without adjustment for uncertainty, except in the bottom two rows where the 5th and 95th percentiles of the uncertainty distribution of total incidence are shown.

Region	Cattle (thousands)	%	Goats (thousands)	%	Pigs (thousands)	%	Sheep (thousands)	%	Buffalo (thousands)	%
	cases/population		cases/population		cases/population		cases/population		cases/population	
China	2806/82,815	3.39	2470/14,360	1.72	10,965/446,463	2.46	2347/136,436	1.72	91/23,272	0.39
India	5912/174,510	3.39	2163/1,25,732	1.72	2/13,770	0.02	1118/64,989	1.72	411/1,05,127	0.39
Rest of Asia	3550/41,997	2.65	2454/1,71,556	1.43	660/86,182	0.76	1174/1,12,863	1.04	174/48,292	0.36
Africa	7403/2,71,493	2.73	4149/2,94,151	1.41	3/26,555	0.01	3269/2,89,907	1.13	0.2/4053	0.01
Europe and Turkey	108/1,40,027	0.08	29/24,660	0.12	0/1,91,537	0.00	121/1,61,070	0.07	0/422	0.02
Middle East	434/13,770	3.15	696/47,262	1.47	0/216	0.00	1644/1,05,778	1.55	4/926	0.39
South America	380/3,42,289	0.11	12/21,220	0.06	0/55,266	0.00	37/76,700	0.05	0/1147	0.01

Total	20,593/1,158,833	1.78	11,973/8,28,175	1.45	11,631/8,19,989	1.42	9709/9,47,743	1.02	680/1,83,237	0.37
5th percentile	22,858/1,158,833	1.97	12,709/8,28,175	1.53	12,346/8,19,989	1.5	10,306/9,47,743	1.08	722/1,83,237	0.39
95th percentile	61,412/1,158,833	5.2	35,706/8,28,175	4.03	34,686/8,19,989	4.2	28,954/9,47,743	3.05	2028/1,83,237	1.11

**Table 4:** Global FMD impact due to vaccination costs and direct, visible production losses in affected stock by region; estimated using variable reported vaccination costs, production losses and uncertain FMD incidence. The variation in total impact is shown (90% range) as well as median estimates. Vaccination costs of between US$0.4 and 3 (most likely US$1) per dose and production losses of between US$100 and 370 (most likely US$100) were used. Outbreaks in free countries were not included.

	Impact US$			
	Production losses	Vaccination	Total	
Region	Median	Median	90% range	Median
China	1.9 billion	2.2 billion	2.5–7 billion	4 billion
India	1.9 billion	0.2 billion	1–4 billion	2.1 billion
Rest of Asia	1.2 billion	70 million	0.7–3 billion	1.3 billion
Africa	2.3 billion	20 million	1–5 billion	2 billion
Europe and Turkey	35 million	20 million	0.03–0.1 billion	0.06 billion
Middle East	0.2 billion	30 million	0.1–0.5 billion	0.22 billion
South America	0.1 billion	0.7 billion	0.5–1.4 billion	0.8 billion
Total	7.6 billion	2.5 billion	6.5–21 billion	11 billion

**Table 5:** Estimated impact of FMD outbreaks in free countries (S.O. = stamping out).

Location	Taiwan^a^	Uruguay^b^	UK^a^	Japan^c^	Rep. Korea^d^
Year	1997	2001	2001	2010	2010–2011
*Costs (US$ millions)*					
Direct costs	254	–	3558	550	2780
Indirect costs	6363	–	5646	N/A	N/A

*Total cost*	6617	700	9204	>550	>2780
Adjusted to value of the US$ in 2011^e^	9450	880	11,600	>568	>2870
As percentage of GDP	−0.64%	N/A	−0.20%	N/A	N/A
Duration (months)	4.5	4	7.5	4	5
Control method	S.O. + Vacc	S.O. + Vacc	S.O.	S.O. + Vacc	S.O. + Vacc
Slaughtered animals	4 million	20,000	6.24 m	2,90,000	3.47 m

a FAO (2002).

b Personal Communication, F. Munzio.

c Muroga et al. (2012).

d ProMED/Yonhap news agency (2011).

e Calculated using contemporary opportunity cost.
